# Supplementary material for: De novo Mutations in Domestic Cat are Consistent with an Effect of Reproductive Longevity on Both the Rate and Spectrum of Mutations
Source: Mol Biol Evol. 2022 Jun 30;39(7):msac147. doi: 10.1093/molbev/msac147 (PMC9290555; doi:10.1093/molbev/msac147)
Supplement: msac147_Supplementary_Data [file msac147_supplementary_data.pdf]

Supplemental Material for

De novo mutations in domestic cat are consistent with an effect of reproductive longevity on both the rate and spectrum of mutations

Richard J. Wang[1], Muthuswamy Raveendran[2,3], R. Alan Harris[2,3], William J. Murphy[4], Leslie A. Lyons[5], Jeffrey Rogers[2,3], and Matthew W. Hahn[1,6]

[1] Department of Biology, Indiana University, Bloomington, IN
[2] Human Genome Sequencing Center, Baylor College of Medicine, Houston, TX
[3] Department of Molecular and Human Genetics, Baylor College of Medicine, Houston, TX
[4] Veterinary Integrative Biosciences, Texas A&M University, College Station, TX
[5] Department of Veterinary Medicine and Surgery, College of Veterinary Medicine, University of Missouri, Columbia, MO
[6] Department of Computer Science, Indiana University, Bloomington, IN

Corresponding author: Richard J. Wang (rjwang@indiana.edu)

Supplemental Results

Comparisons of the mutation spectrum to the rare variant spectrum

We compared the cat mutation spectrum to the spectrum of rare polymorphisms from a published study of cat variation. To calculate the rare variant spectrum, we considered variants from the 99 Lives dataset (Buckley et al. 2020) at frequencies less than 5% in the folded frequency spectrum. We excluded singletons to reduce potential sequencing errors. Table S1 shows a comparison of this calculated spectrum to the mutation spectrum. A>G transitions are significantly overrepresented while A>T transversions are significantly underrepresented (both $p < 0.05$) in the rare variant spectrum relative to the mutation spectrum.

We performed the same comparison in humans, using extremely rare variants from Carlson et al. (2018) to calculate the polymorphism spectrum and mutations from Jónsson et al. (2017) to calculate the mutation spectrum. There is more power to detect differences with the larger number of mutations in the human dataset. We find that all but the proportion of A>T transversions are significantly different at the $p < 0.05$ level between the rare variant spectrum and the mutation spectrum for humans (Table S1).

Table S1. Comparison of mutation and variant spectra in cat and human

	**mutation class (percent)**					
**cat spectrum**	A>C	A>G	A>T	C>A	C>G	C>T
mutation	6.44	18.88	11.59	9.01	6.44	47.64
rare variant	7.38	27.31	5.70	8.54	7.48	43.59
**human spectrum**						
mutation	7.04	26.81	6.80	7.59	9.60	42.15
rare variant	7.30	27.23	6.98	10.19	8.81	39.49

Supplemental Figures

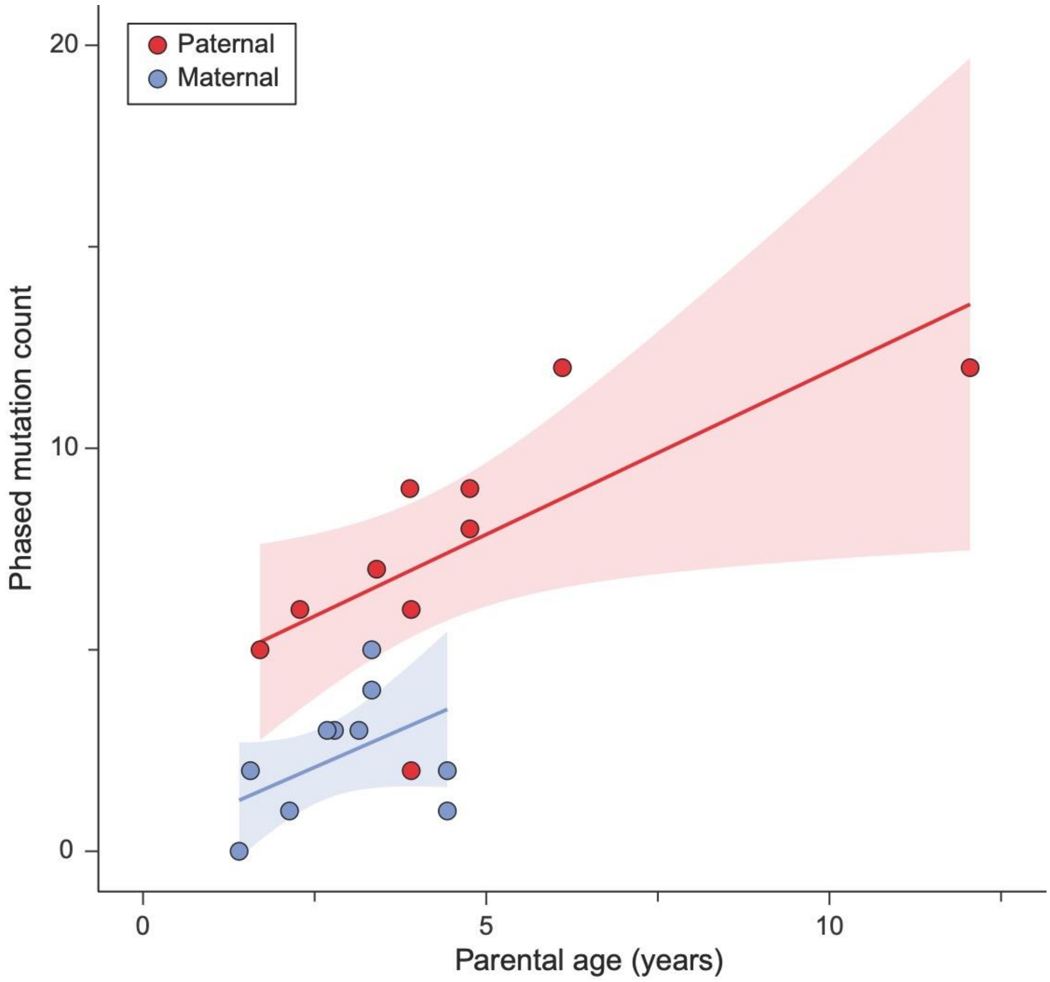

Figure S1. Phased mutation counts with parental age

The number of phased mutations from ten trios of the domestic cat with a maternal (blue) and paternal origin (red), as identified by read-pair phasing. There is a positive relationship between the number of phased mutations and increasing parental age, though fewer maternal mutations limit the significance of its p-value (Poisson regression, paternal $p = 0.032$, maternal $p = 0.116$). Shaded areas show regression 95% CI.

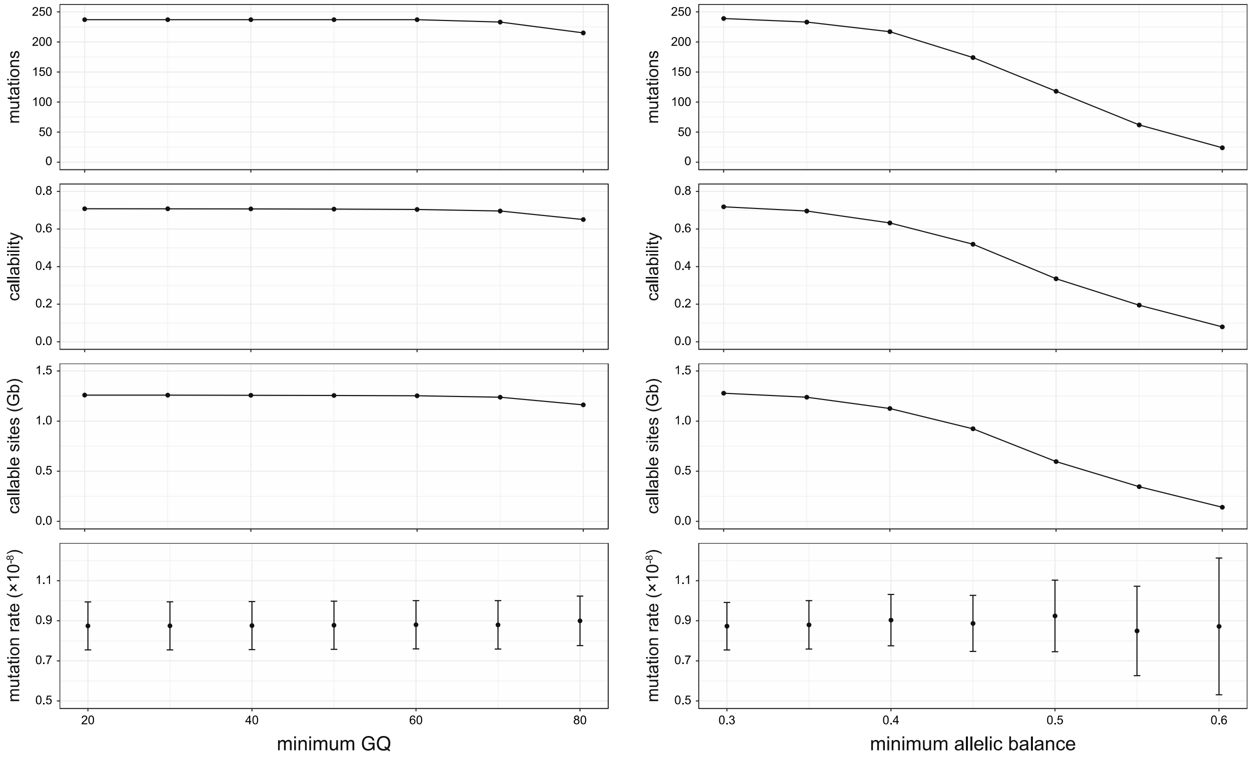

Figure S2. Mutation rates estimated with increasing filter stringency

The number of mutations and the callable size of the genome decline with increasing filter stringency for (left) minimum genotype quality (GQ), while maintaining allelic balance > 0.35, and (right) minimum allelic balance, while GQ > 70. Error bars show 95% CI on estimates of the per generation, per bp rates under a Poisson model.

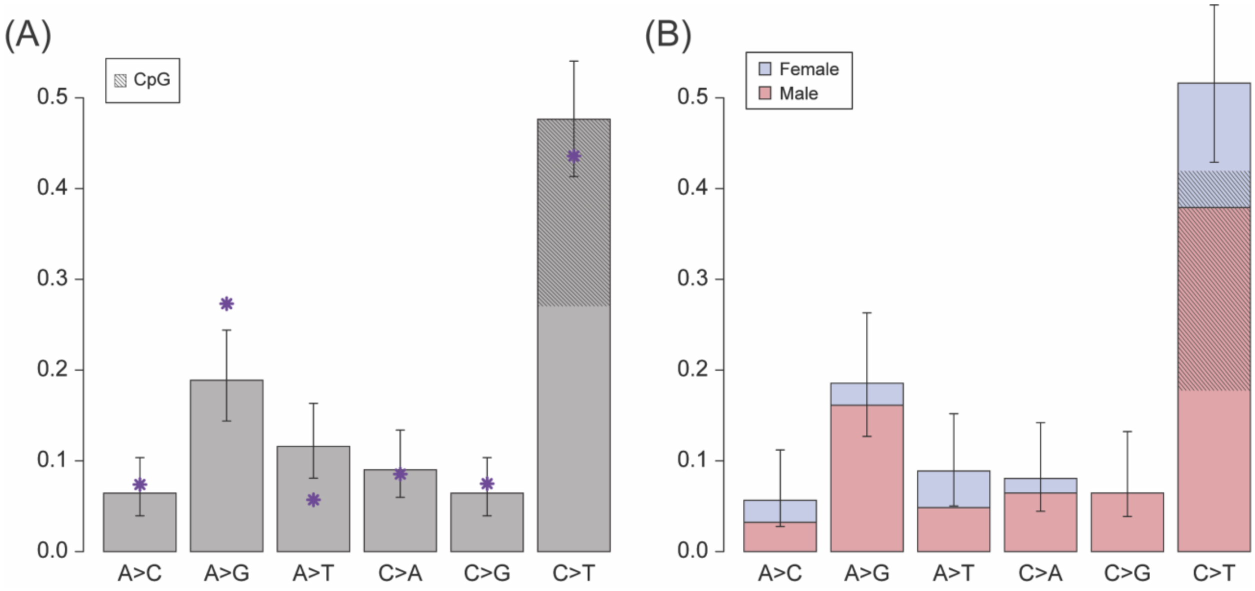

Figure S3. Cat mutation spectrum

(A) Proportion of each mutation class from mutations detected in all 11 trios. Asterisks (purple) show a comparison to the polymorphism spectrum for each mutation class (see Supplemental Results). (B) Proportion of each mutation class from only phased mutations. Shaded regions show the proportion of mutations occurring at CpG sites. 55% of phased C>T mutations occurred at CpG sites: 47% of these were of paternal origin and 8% were of maternal origin. Error bars show binomial 95% CI (Wilson score interval) for respective classes.

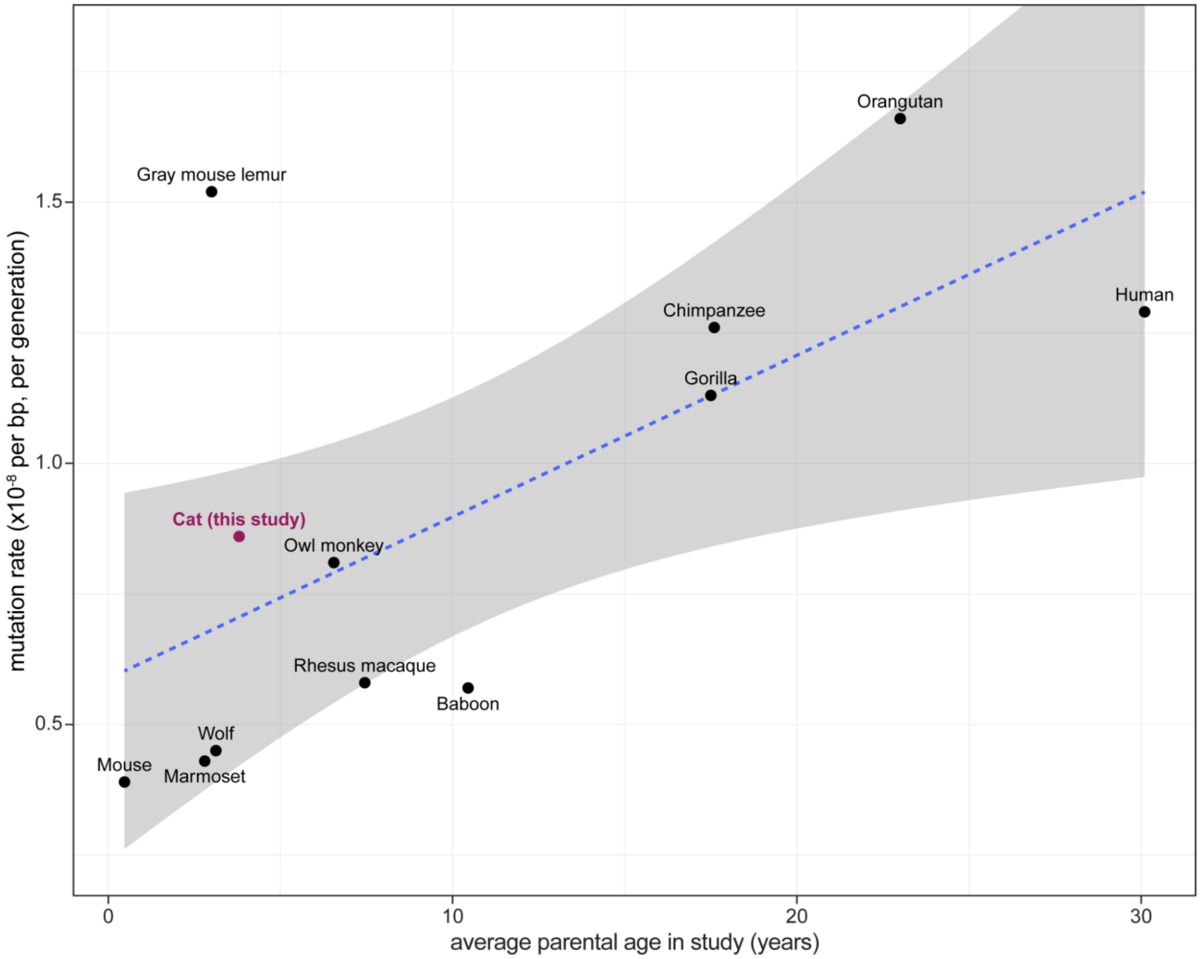

Figure S4. Mutation rate per generation and parental age in mammals

Comparison of the per-generation mutation rate from the domestic cat (red) to rates from other pedigree studies in mammals. There is a significant relationship between the mutation rate and average parental age of animals used in each study ($p = 0.02$). Because these studies use different sampling approaches, we show a point estimate at the average age of animals used in each study to make the comparison. The blue dotted line shows a linear regression of the per-generation mutation rate with parental age from all presented estimates (95% CI in gray). References for each of these estimates:

Mouse, Lindsay et al. (2019); *Wolf*, Koch et al. (2019); *Marmoset*, Yang et al. (2021); *Gray mouse lemur*, Campbell et al. (2021); *Owl monkey*, Thomas et al. (2018); *Rhesus macaque*, Wang et al. (2020); *Baboon*, Wu et al. (2020); *Chimpanzee*, Besenbacher et al. (2019); *Gorilla*, Besenbacher et al. (2019); *Orangutan*, Besenbacher et al. (2019); *Human*, Jónsson et al. (2017).

References

Besenbacher S, Hvilsom C, Marques-Bonet T, Mailund T, Schierup MH. 2019. Direct estimation of mutations in great apes reconciles phylogenetic dating. *Nature Ecology & Evolution* 3:286–292.

Buckley RM, Davis BW, Brashear WA, Farias FHG, Kuroki K, Graves T, Hillier LW, Kremitzki M, Li G, Middleton RP, et al. 2020. A new domestic cat genome assembly based on long sequence reads empowers feline genomic medicine and identifies a novel gene for dwarfism. *PLOS Genet.* 16:e1008926.

Campbell CR, Tiley GP, Poelstra JW, Hunnicutt KE, Larsen PA, Lee H-J, Thorne JL, dos Reis M, Yoder AD. 2021. Pedigree-based and phylogenetic methods support surprising patterns of mutation rate and spectrum in the gray mouse lemur. *Heredity* 127:233–244.

Carlson J, Locke AE, Flickinger M, Zawistowski M, Levy S, Myers RM, Boehnke M, Kang HM, Scott LJ, Li JZ, et al. 2018. Extremely rare variants reveal patterns of germline mutation rate heterogeneity in humans. *Nature Communications* 9:3753.

Jónsson H, Sulem P, Kehr B, Kristmundsdottir S, Zink F, Hjartarson E, Hardarson MT, Hjorleifsson KE, Eggertsson HP, Gudjonsson SA, et al. 2017. Parental influence on human germline *de novo* mutations in 1,548 trios from Iceland. *Nature* 549:519–522.

Koch EM, Schweizer RM, Schweizer TM, Stahler DR, Smith DW, Wayne RK, Novembre J. 2019. *De novo* mutation rate estimation in wolves of known pedigree. *Molecular Biology and Evolution* 36:2536–2547.

Lindsay SJ, Rahbari R, Kaplanis J, Keane T, Hurles ME. 2019. Similarities and differences in patterns of germline mutation between mice and humans. *Nature Communications* 10:4053.

Thomas GWC, Wang RJ, Puri A, Harris RA, Raveendran M, Hughes DST, Murali SC, Williams LE, Doddapaneni H, Muzny DM, et al. 2018. Reproductive longevity predicts mutation rates in primates. *Current Biology* 28:3193–3197.

Wang RJ, Thomas GWC, Raveendran M, Harris RA, Doddapaneni H, Muzny DM, Capitanio JP, Radivojac P, Rogers J, Hahn MW. 2020. Paternal age in rhesus macaques is positively associated with germline mutation accumulation but not with measures of offspring sociability. *Genome Research* 30:826–834.

Wu FL, Strand AI, Cox LA, Ober C, Wall JD, Moorjani P, Przeworski M. 2020. A comparison of humans and baboons suggests germline mutation rates do not track cell divisions. *PLOS Biology* 18:e3000838.

Yang C, Zhou Y, Marcus S, Formenti G, Bergeron LA, Song Z, Bi X, Bergman J, Rousselle MMC, Zhou C, et al. 2021. Evolutionary and biomedical insights from a marmoset diploid genome assembly. *Nature* 594:227–233.
